# Ontario Veterinary Association, Annual Meeting

**Published:** 1894-01

**Authors:** 


### ANNUAL MEETING OF THE ONTARIO VETERINARY ASSOCIATION.

The annual meeting of this association was held in the Veterinary College, Toronto, on Thursday, Dec. 21st, 1893.

The President, Mr. John Wende, V.S., of Buffalo, N. Y., in the chair.

In his opening address Mr. Wende spoke with much feeling of his gratification at being placed in the honorable position he occupied, and he impressed on the members present the advantages of associations of the various professions, and indeed of all the callings in life, and of their meeting for mutual improvement and discussion.

The Secretary's, Registrar's, Treasurer's, and Auditor's reports were then received and adopted.

Mr. John Wende, read an interesting paper on pyo-septhæmia, commonly called "joint ill," in foals. He mentioned the different names by which it was known, and the various views as to its pathology, which had been held. He said that Bollinger, in 1873, first recognized it as omphalo-phlebitis. He fully described the symptoms, also the post-mortem appearances produced by emboli in the capillaries of the viscera. He mentioned that "pervious urachus," though frequent, was not invariably present. It is a very serious disease. He also gave the line of treatment he adopted.

Messrs. Crowforth, W. J. Wilson and others, took part in the discussion that followed.

In a discussion in which Mr. C. Elliott, Mayor Lloyd, Mr. Quinn and others took part, it was ultimately suggested that the Council of Acts should send representatives to attend at the examinations of the Ontario Veterinary College.

A discussion then took place on certain parties advertising as teaching veterinary dentistry, and granting diplomas as Veterinary Dentists. And a resolution was passed that a committee be formed to frame a resolution strongly condemning such parties for issuing their so-called Veterinary Dental Diplomas. The resolution to be presented to the Provincial Secretary and to the County Attorney, who were to be interviewed on the subject.

Moved by Mr. O'Neil, seconded by Mayor Lloyd, and carried, that a motion of condolence be forwarded to the widow of the late Mr. Hand of Alliston, an old and respected member of this association, expressing deep feelings of sympathy with her in her late sad bereavement.

The question of contagious pleuro pneumonia in cattle then came up. and a resolution was moved by Mr C. Elliott, seconded by Mr. W. J. Wilson, "That no contagious pleuro-pneumonia exists at the present time, neither has that disease ever made its appearance in the Province of Ontario."

This was carried unanimously.

Mr. John Wende gave a short account of his attendance at the United States Veterinary Congress at Chicago.

Moved by Mr. W J. Wilson, seconded by Mr. A. Crowforth, and carried, that all the directors read a paper at the next meeting of the association.

The following new members were duly elected: Mr. W. C. McGuire, D V.S., of Shawville, Quebec; Mr. A. Crowforth, V.S., of Lockport, N.Y.; and Mr S. T. Holder, V.S., of Mount Albert, Ont.

The officers for the ensuing year are: Mr. W. Burns, V.S., King, President; Mr. G L. Robson, V.S., Manchester, First Vice-President; Mr. H. Hopkins, V.S., Green River, Second Vice-President; Mr. C. H. Sweetapple, V.S., Toronto, Secretary· Mr. W. Cowan, V S., Galt, Treasurer.

*Directors* —J. Wende, D. Hamilton, J. F. Quinn, W Gibb, W. J. Wilson, S. T. Holder, A. Crowforth, and W. Steele.

*Auditors.*—Messrs. C. Elliott and J D. O'Neil.

Messrs. J. H. Wilson and J. D. O'Neil were appointed representatives to the Western Fair Association.

Mr. W. Cowan was appointed representative to the Central Farmers' Institute.

